# Cyclopædia of the Practice of Medicine

**Published:** 1877-02

**Authors:** 


Cyclopædia of the Practice of Medicine. Edited by Dr. H. Von Ziemssen, Professor of Clinical Medicine in Munich, Bavaria. Publishers, Wm. Wood & Co: New York. 1877.

Volumes six and seven of this work have just been received. Volume six is devoted to diseases of the circulatory system, together with chapters on *whooping cough, diseases of the lips and cavity of the mouth, and of the soft palate.* The names of several very prominent gentlemen are mentioned as contributors in the completion of this volume. Volume seven considers diseases of the chylopoietic system, together with *diseases of the naso-pharyngeal cavity and pharynx, laryngetis, phlegmoosa, pericarditis laryngea, ulcerations, tumors, and neuroses of the larynx.*

It is not necessary to attempt to detail the points of interest in these two books. Already the work has attained quite a high reputation, and everyone expects, very reasonably too, points of interest and profit to be presented. As has often been said, this collection, in all its volumes, will make a library of itself, and no enterprising physician should be without it.

